# Obliteration of the Inferior Cava, Iliacs, Crural, and Other Veins

**Published:** 1831

**Authors:** 


Obliteration of the Inferior Cava, Iliacs, Crural, and other Veins. By M. Reynauld, M.D.

[Hôtel Dieu.]

*Case.* The patient was 40 years of age, and had had a fracture of the fibula eight months prior to his entering the hospital. One month before his admission he had experienced a fall, and contused the right side of the chest, some days after which he complanied of pain in the inner and lower part of the right thigh, extending up to the groin, with swelling of the whole limb. There was nothing of the kind in the other member, except simply a swelling, which increased rapidly. On the 9th May (the day after coming into hospital) there was œdema of both legs; but much greater in the left than in the right. There was neither redness, nor hardness—and there was now but little pain. For two days past there was immobility (not paralysis) of the left limb. Near the groin of this side, there were three red lines or bands. The veins of both flanks were very much distended. Some œdema in the lower part of the abdomen on the left side. The pulse was 100, and the beating of the heart limited to the usual space. Very little thirst —anorexia. Venesection. The blood was buffed. 10th. A rigor, followed by heat and perspiration. This recurred on the 11th, after another bleeding. 12th. The abdominal veins are still more distended, and it was observed that the current of the circulation in them was reversed. After this the fever subsided—the pain was felt in the left hypochondrium and in the left side of the chest. During the three succeeding months the œdema of the lower extremities diminished considerably in the right side; but not so much in the left. By the middle of July, however, it had subsided in both extremities. The epigastric vessels of the right side became more distended than those of the *left* —but ultimately they both diminished. Cough which had existed for some time ceased, as did the pain of the side. All feverishness disappeared; but the appetite never returned. The strength returned very slowly, and the patient could only go upon crutches. He became tired of the hospital, and removed home for a while; but ultimately returned to the hospital, and died there, with his legs moderately infiltrated.

*On dissection,* it was observed that the cava inferior diminished rapidly from the junction of the renal veins to its termination in the iliacs. Where the right iliac vein is given off, the cava was obstructed by a clot of fibrine which adhered to the parietes of the vessel. This clot extended up to the origin of the renal vessels; and also obstructed the iliac veins on both sides. Below this obstruction the vessels appeared like solid cords. The same was observed of the crural veins, the saphenæ, and the popliteal; as well as all the principal venous trunks of the lower extremities. In the abdomen, the only vessels that had enlarged were the spermatic veins. They were as large as the ovarian veins in the last month of pregnancy. Those of the right side entered the cava above where the obstruction existed. The left spermatics emptied themselves into the corresponding renal vein. The circumflexa ilii and one of the lumbar veins were larger than natural. On the parietes of the abdomen, also, there was a network of vessels exceedingly multiplied. The left renal vein, the splenic vein, and a branch of the vena portæ were obstructed in the same manner as the inferior cava. There were some fibrinous obstructions in the aorta; the lungs were cavernous—and there was effusion in the cavity of the right pleura.—*Journ. Hebdomadaire.*

